# Cerebral venous sinus thrombosis presenting with diplopia in pregnancy: a case report

**DOI:** 10.1186/1752-1947-6-336

**Published:** 2012-10-03

**Authors:** Yusoff Munira, Zakariah Sakinah, Embong Zunaina

**Affiliations:** 1 Department of Ophthalmology, Hospital Raja Perempuan Zainab II, 15886 Kota Bharu, Kelantan, Malaysia; 2 Department of Ophthalmology, School of Medical Sciences, Universiti Sains Malaysia, 16150 Kubang Kerian, Kelantan, Malaysia

**Keywords:** Cerebral venous sinus thrombosis, Diplopia, Pregnancy

## Abstract

**Introduction:**

Cerebral venous sinus thrombosis is a rare condition. The most frequent symptoms and signs are headache, focal seizures with or without secondary generalization, unilateral or bilateral paresis and papilledema. We report a case of transverse sinus and superior sagittal sinus thrombosis that presented with diplopia in a pregnant woman.

**Case presentation:**

A 34-year-old Malay woman, gravida 3 para 2 at 8 weeks of pregnancy, was admitted for hyperemesis gravidarum, presented with sudden onset of diplopia, blurring of vision and headache. A magnetic resonance scan of her brain showed the presence of cerebral edema with no space occupying lesion, but magnetic resonance venography ultimately revealed right transverse sinus and superior sagittal sinus thrombosis. The patient was treated with anticoagulation for 1 year, after which the patient recovered fully.

**Conclusion:**

Due to its diverse and varied neurological presentation, cerebral venous sinus thrombosis should be considered in almost any brain syndrome.

## Introduction

Cerebral venous sinus thrombosis is a rare condition and its clinical presentation is extremely variable and life-threatening [1,2]. The most frequent symptoms and signs are headache (95%), focal seizures with or without secondary generalization (47%), unilateral or bilateral paresis (43%) and papilledema (41%) [3]. Patients who are at risk of developing venous thrombus formation are those with hypercoagulable states, dehydration, adjacent infectious processes, low cerebral blood flow, oral contraceptives, hormone replacement therapy, pregnancy, and puerperium [1,2,4].

## Case presentation

A 34-year-old Malay woman, gravida 3 para 2 at 8 weeks of pregnancy, presented with nausea and vomiting for 4 days’ duration. She was admitted into our prenatal ward and was diagnosed as hyperemesis gravidarum. After 3 days in the ward, she developed sudden onset of diplopia associated with blurring of vision in both eyes and fronto-temporal headache.

It was not associated with fever or rash. There was no history of facial or sinus infection and no history of recent head trauma. She was completely well previously with no significant medical history. She had never experienced similar symptoms during her previous pregnancy and there was no family history of similar conditions noted.

She was alert and conscious and her vital signs were stable. The visual acuity in her right eye was 6/24 and improved to 6/6 with pinhole. Her left eye visual acuity was 6/24 and improved to 6/9 with pinhole. Extraocular muscle examination showed bilateral sixth cranial nerve palsy. Anterior segment examination was normal. Fundus examination showed bilateral papilledema. Other cranial nerves were normal. There were no peripheral neurological signs noted.

**Figure 1:**
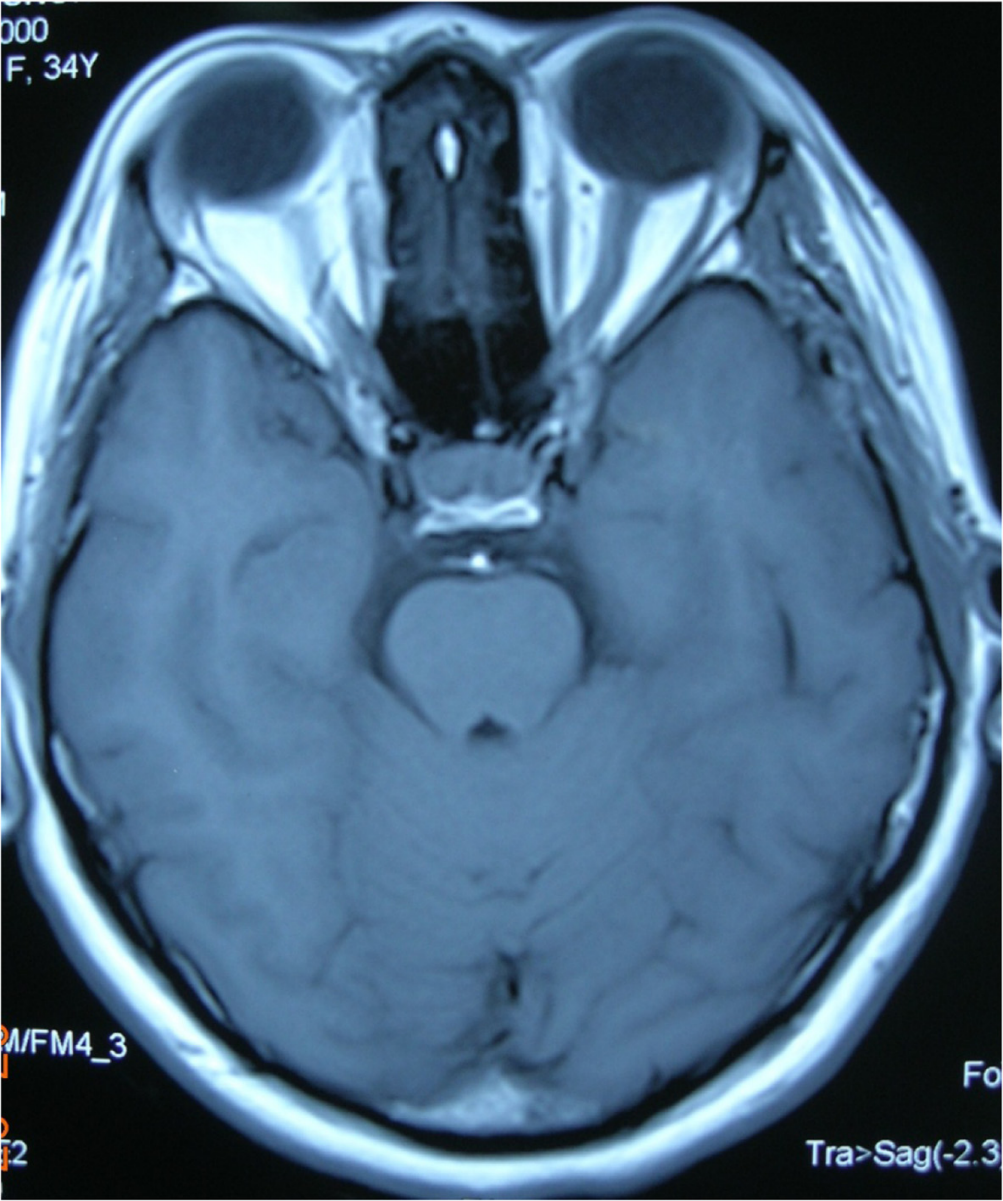
Magnetic resonance scan of the orbit and brain showed cerebral edema with normal orbits and no space occupying lesion.

**Figure 2:**
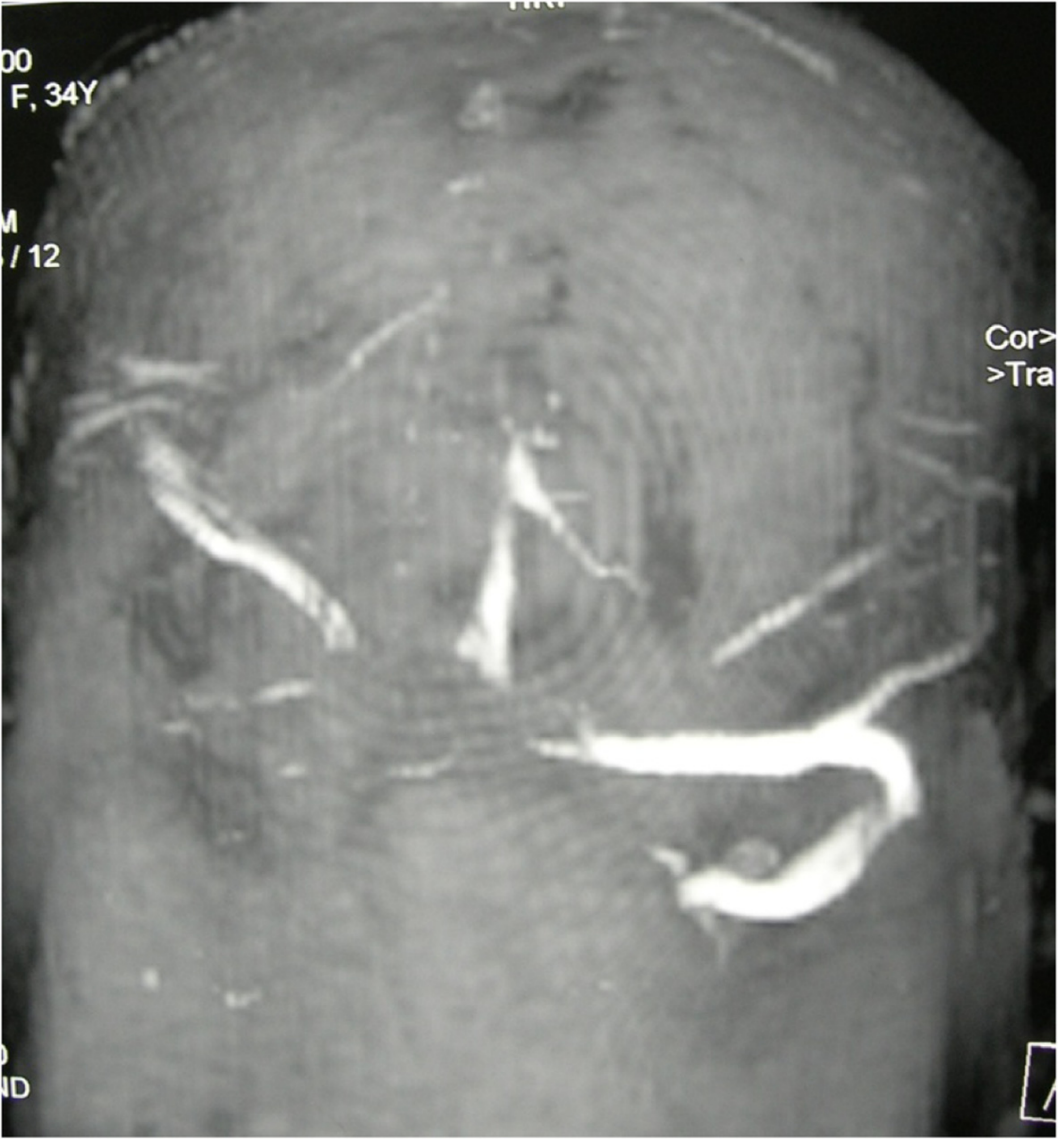
Magnetic resonance venography showing absence of signal of part of right transverse sinus and superior sagittal sinus.

A magnetic resonance scan of the patient’s brain showed presence of cerebral edema with normal orbits and no space occupying lesion (Figure1). Magnetic resonance venography (MRV) revealed right transverse sinus and superior sagittal sinus thrombosis (Figure2). Her coagulation profile and blood investigations for connective tissue disorder were normal, and echocardiogram was also normal.

**Figure 3:**
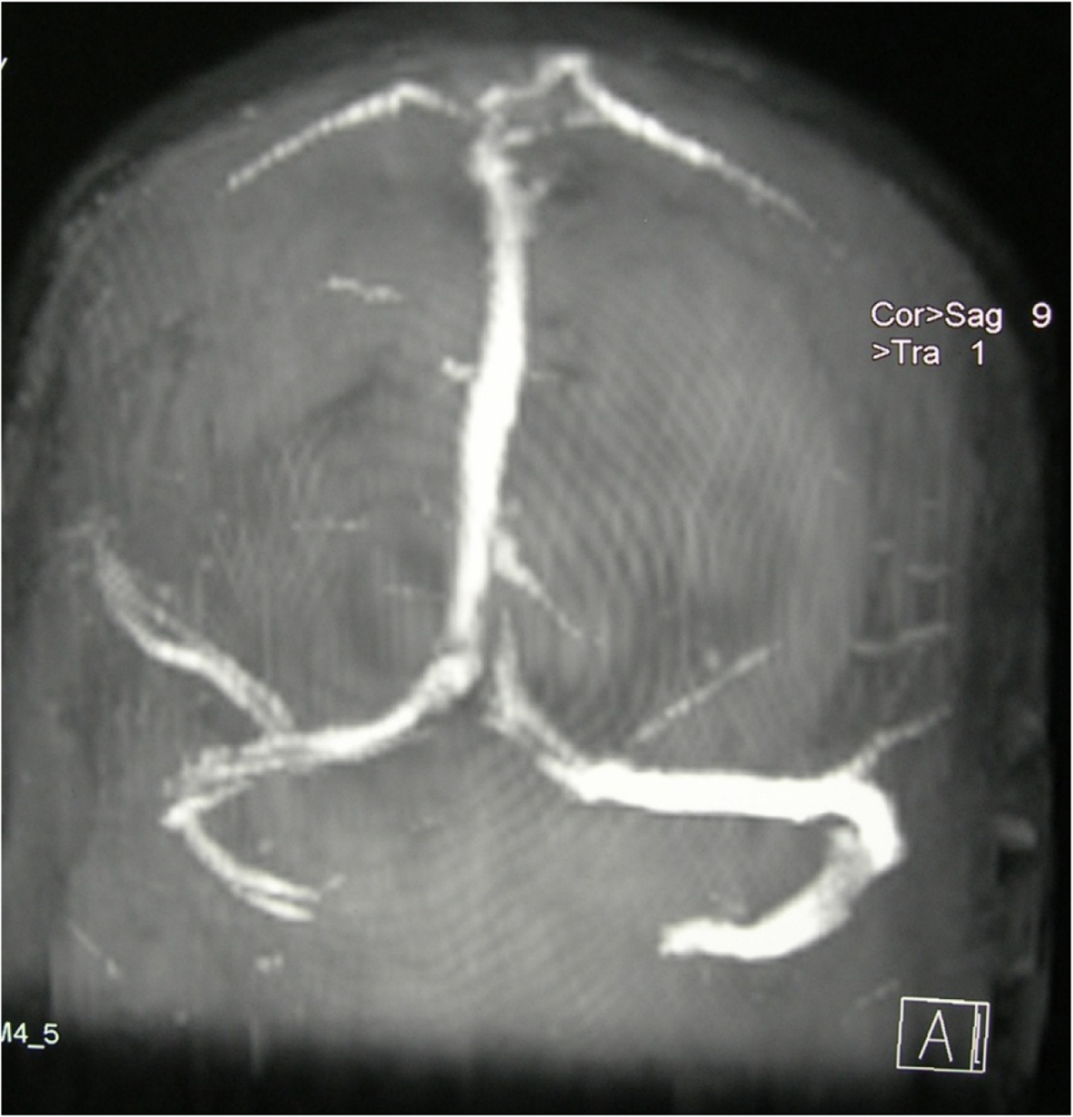
Repeat magnetic resonance venography at 1 month after anticoagulation showed visualized flow signal of right transverse and superior sagittal sinus.

Our Neurology Team diagnosed cerebral venous sinus thrombosis. The patient was treated with intravenous heparin for 1 week followed by subcutaneous heparin for 3 weeks. Diplopia and headache had gradually resolved and her visual acuity had improved after 1 month of anticoagulation. MRV was repeated at 1 month after the initiation of treatment and showed resolved cerebral venous sinus thrombosis (Figure3). Because cerebral venous sinus thrombosis poses a high risk of thromboembolism in this patient and furthermore no other safer drug was available, warfarin was subsequently initiated. Warfarin was started at 13 weeks’ period of amenorrhea and she was treated with long-term warfarin for 11 months. The dose of warfarin was adjusted carefully based on her International Normalised Ratio (INR) reading with the aim to maintain her INR between 2 and 3. The patient had given birth uneventfully at term. The baby was born at 38 weeks period of amenorrhea via spontaneous vaginal delivery with birth weight of 2.8kg, without any complication and there was no identifiable syndrome noted.

## Discussion

Cerebral venous sinus thrombosis is a rare and potentially deadly condition. It occurs 10 to 13 times more often during the puerperium than during pregnancy and is also very rare during the first trimester. The most common postulated mechanism on how cerebral venous sinus thrombosis can affect a pregnant woman is hypercoagulable state brought about by the pregnancy itself and further aggravated by dehydration and anemia [1].

Once the thrombus is formed, it may enlarge and cause venous congestion. This will lead to cerebral edema with mass effect and a resultant increase of intracranial pressure. If the thrombus is left untreated, the intracranial pressure will continue to rise and the vascular supply is compromised. Finally ischemia will ensue.

According to The International Study on Cerebral Vein and Dural Sinus Thrombosis, the most commonly affected site is the transverse sinus, followed by superior sagittal sinus and straight sinus [5]. Other less common sites are the cortical vein, jugular vein and internal cerebral vein. In most patients, thrombosis occurs in more than one sinus.

The mode of onset is highly variable, which can be from sudden to progressive over weeks, so it can mimic other conditions such as tumor, stroke, or benign intracranial hypertension. Headache, focal deficits, seizures, disorders of consciousness, and papilledema, which can present in isolation or in association, are the most frequent signs [6,7]. Diplopia is not a common presenting symptom of cerebral venous sinus thrombosis [8]. If the intracranial pressure is quite high, a sixth cranial nerve palsy may develop. Usually it presents as a false localizing sign, but it may also indicate extension of thrombus into the inferior petrosal sinus [9].

According to the guidelines of the European Federation of Neurological Societies, the first line treatment for cerebral venous and dural sinus thrombosis is antithrombolysis. The rationale for its use is to favor spontaneous thrombus resolution and to recanalize the occluded vein or sinus, to avoid thrombus propagation, to treat underlying prothrombotic condition and to prevent complications such as pulmonary thromboembolism. Treatment is usually started with dose-adjusted intravenous heparin between 3000 and 5000 international units or body-weight-adjusted subcutaneous low-molecular-weight heparin, until the patient stabilizes. Treatment for this acute phase is followed by oral anticoagulation with warfarin to prevent recurrence and thrombosis in other parts of the body.

The optimal duration of oral treatment varies, but the accepted practice is 3 months for idiopathic cases, between 3 and 6 months for cases related to pregnancy or oral contraceptive use, and between 6 and 12 months in patients with hereditary thrombophilia [6,10]. This treatment is aimed for INR of 2 or 3. Some articles suggest catheter-guided local thrombolysis in patients who deteriorate despite adequate anticoagulation.

The prognosis is quite good in 80% of patients with obstetric causes [1] and has a mortality rate of between 5 and 10% [1,5,11]. Our patient was treated with anticoagulation for 1 year, after which the patient had recovered fully and had given birth uneventfully at term.

## Conclusions

Due to its diverse and varied neurological presentation, cerebral venous sinus thrombosis should be considered in almost any brain syndrome.

## Consent

Written informed consent was obtained from the patient for publication of this case report and any accompanying images. A copy of the written consent is available for review by the Editor-in-Chief of this journal.

## Competing interests

The authors declare that they have no competing interests.

## Authors’ contributions

YM examined and evaluated the patient and wrote the manuscript. ZS examined and evaluated the patient. EZ edited the manuscript. All authors read and approved the final manuscript.
